# Exploring the relationship between uric acid-to-HDL cholesterol ratio and vascular calcification: evidence from clinical, genetic, and network-based analyses

**DOI:** 10.3389/fcvm.2026.1877627

**Published:** 2026-08-12

**Authors:** Xinyi Li, Yang Cao, ZiQin Xiao, Jianhua Luo, Lin Wang, Yan Zhang, Qingchun Liang, Bing Liang

**Affiliations:** 1 Department of Anesthesiology, The Affiliated Brain Hospital of Guangzhou Medical University (Guangzhou Huiai Hospital), Guangzhou, China; 2 Department of Anesthesiology, Guangzhou Red Cross Hospital of Jinan University, Guangzhou, China; 3 Department of Anesthesiology, The Third Affiliated Hospital of Southern Medical University, Guangzhou, China; 4 Department of Anesthesiology, Linzhi People’s Hospital, Linzhi, China; 5 The First School of Clinical Medicine, Southern Medical University, Guangzhou, China

**Keywords:** aortic arch calcification, Mendelian randomization, network analysis, uric acid to high-density lipoprotein cholesterol ratio, vascular calcification

## Abstract

**Background:**

Vascular calcification (VC) is a multifactorial process associated with adverse cardiovascular outcomes. The uric acid to high-density lipoprotein cholesterol ratio (UHR) has been proposed as a composite metabolic biomarker that reflects the balance between pro-oxidative and anti-inflammatory processes. However, its association with VC and the underlying mechanisms remain incompletely understood.

**Methods:**

This study integrated clinical association analysis, Mendelian randomization (MR), and network-based approaches. A retrospective cross-sectional analysis was conducted in 627 clinically stable cardiology inpatients, with aortic arch calcification (AoAC) assessed using chest radiographs. Multivariable logistic regression and restricted cubic spline analyses were used to examine the association between UHR and AoAC. Two-sample MR was performed to assess the potential causal effects of uric acid (UA) and high-density lipoprotein cholesterol (HDL-C) on abdominal aortic calcification (AAC), a macrovascular calcification phenotype. Network-based analysis was conducted to explore potential molecular mechanisms.

**Results:**

Higher UHR levels were significantly associated with greater AoAC severity, which remained after adjusting for standard cardiovascular risk factors. MR analysis showed no significant genetic association between genetically predicted UA and AAC, whereas genetically predicted HDL-C showed an inverse association with AAC in the primary IVW analysis, but this was not consistently supported across sensitivity methods. Network-based analyses identified enrichment in inflammation- and remodeling-related pathways, including MAPK and PI3K–Akt signaling, with key hub targets such as TNF, AKT1, and EGFR.

**Conclusion:**

Higher UHR levels were associated with greater VC burden in clinically stable cardiology inpatients. The clinical, genetic, and network-based findings provide complementary evidence suggesting that UHR may reflect the combined influence of multiple metabolic and inflammatory processes associated with VC, rather than the direct effect of any single component.

## 1 Introduction

Vascular calcification (VC) is currently recognized as an active, cell-regulated process characterized by the osteogenic transformation of vascular smooth muscle cells (VSMCs) (1). Although traditionally regarded as a degenerative phenomenon, accumulating evidence indicates that VC is a tightly regulated process influenced by aging, metabolic disorders, and genetic susceptibility (2). Clinically, VC is a hallmark of vascular aging and is independently associated with adverse cardiovascular outcomes (2). Among its imaging manifestations, aortic arch calcification (AoAC), detected on routine chest radiographs, serves as a practical and cost-effective imaging marker of systemic macrovascular calcification. Increasing evidence has demonstrated that AoAC correlates closely with coronary and extracoronary calcific burden and cardiovascular risk (3, 4). These observations underscore the importance of identifying metabolic biomarkers associated with VC and its progression.

Redox imbalance and chronic low-grade inflammatory signaling within the vascular wall are increasingly recognized as key contributors to VC progression (5, 6). Uric acid (UA), the terminal metabolite of purine catabolism, has been implicated in oxidative stress, inflammatory signaling, endothelial dysfunction, and osteogenic differentiation of VSMCs, all of which are closely linked to the development of VC (7, 8). Elevated UA is also closely associated with metabolic disorders such as hypertension, insulin resistance, obesity, and chronic kidney disease (9). These metabolic abnormalities have also been implicated in the development and progression of VC (10, 11). Conversely, high-density lipoprotein cholesterol (HDL-C) plays a crucial role in protecting blood vessels by maintaining endothelial health, facilitating cholesterol removal, and reducing vascular inflammation (12). These protective effects are mediated through multiple mechanisms, including antioxidant and anti-inflammatory activities (13).

Given the contrasting biological effects of UA and HDL-C, the uric acid to high-density lipoprotein cholesterol ratio(UHR) has emerged as a combined biomarker reflecting the balance between metabolic stress and vascular protection. By combining a pro-oxidative metabolic factor with an anti-inflammatory lipoprotein component, UHR may provide a more comprehensive assessment of metabolic-inflammatory status than either biomarker alone. Recent studies have associated elevated UHR with VC as well as a range of adverse cardiovascular and metabolic outcomes, including postoperative acute kidney injury in patients undergoing coronary artery bypass grafting, coronary slow flow phenomenon, and unfavorable functional outcomes following acute ischemic stroke (14–17) These findings suggest that UHR may better capture the complex metabolic and inflammatory milieu underlying vascular remodeling and calcification.

However, whether UHR reflects the burden and severity of VC remains unclear. Because VC is a systemic pathological process, different but related phenotypes may be used to represent complementary aspects of the same disease continuum. Although both UA and HDL-C have been individually associated with VC in epidemiological studies (18, 19), whether their combined metabolic profile, represented by UHR, provides additional clinical or biological insight remains unclear. Moreover, the molecular mechanisms through which UHR-related metabolic imbalance may influence VC have not been fully elucidated. Therefore, this study integrated clinical association analysis, Mendelian randomization (MR), and network-based analysis to investigate the relationship between UHR and VC from complementary clinical, genetic, and mechanistic perspectives. Together, these complementary analytical layers may provide a more comprehensive understanding of the metabolic-inflammatory processes underlying VC and the potential translational value of UHR as a composite metabolic biomarker.

## 2 Materials and methods

### 2.1 Study design

This study integrated clinical association analysis, MR analysis, and mechanistic exploration to evaluate the relationship between the UHR and VC. In the clinical analysis, AoAC assessed from chest radiographs was used as a clinically accessible imaging phenotype to investigate the association between UHR and VC. In the genetic analysis, MR was performed using abdominal aortic calcification (AAC) derived from genome-wide association study (GWAS) datasets as a genetic phenotype of macrovascular calcification to investigate genetic evidence related to the individual components of UHR, namely UA and HDL-C. In addition, network-based analyses were conducted to systematically explore potential molecular targets and signaling pathways involved in VC, thereby providing exploratory mechanistic evidence.

Overall, this study addressed a question through three complementary analytical layers, including clinical association, genetic investigation, and mechanistic exploration. Rather than providing replication of an identical exposure–outcome relationship, these analytical layers were designed to offer complementary information regarding clinical association, component-level genetic evidence, and potential biological mechanisms, thereby facilitating a more comprehensive understanding of the relationship between UHR and VC.

### 2.2 Clinical association analysis

#### 2.2.1 Study population

The clinical part of this research was performed as a retrospective cross-sectional analysis at the Department of Cardiology, Guangzhou Red Cross Hospital of Jinan University. Eligibility screening was conducted for consecutive inpatients admitted between September 1, 2024, and August 31, 2025. To minimize the influence of acute physiological stress on metabolic biomarkers, the study population was restricted to patients in a clinically stable phase of cardiovascular management, including individuals with chronic cardiovascular conditions such as essential hypertension and stable coronary artery disease. Clinical stability was defined as the absence of acute cardiovascular events, worsening cardiovascular symptoms, or emergency cardiovascular hospitalization or intervention within the preceding three months.

Eligible participants were aged 40–80 years and had available chest radiographs as well as complete fasting laboratory measurements obtained within 24 h of admission. Patients with acute heart failure or acute myocardial infarction within the preceding three months, active malignancy, autoimmune diseases, acute infection, documented use of urate-lowering medications, or incomplete clinical or imaging data were excluded. The final sample consisted of all eligible patients identified during the study period.

Approval for the study protocol was granted by the Ethics Committee of Guangzhou Red Cross Hospital (Approval No. 2025-243-02), with informed consent waived due to the study's retrospective and anonymized design.

#### 2.2.2 Data collection and definitions

Data on demographic characteristics, lifestyle factors, comorbidities, medication history, and lab parameters were extracted from the electronic medical records. Comorbidities, including hypertension, diabetes mellitus, CHD, dyslipidemia, arrhythmia, HF, CKD, and stroke, were identified based on physician-documented diagnoses recorded in the electronic medical records. Demographic characteristics included age, sex, and body mass index (BMI), which was calculated as weight in kilograms divided by the square of height in meters. Lifestyle factors included smoking and drinking status. Medication history included the use of antihypertensive agents, glucose-lowering medications, statins, and antiplatelet agents. Laboratory parameters included white blood cell count, hemoglobin, platelet count, fasting plasma glucose, creatinine, UA, triglycerides, total cholesterol, HDL-C, and low-density lipoprotein cholesterol (LDL-C). Estimated glomerular filtration rate (eGFR) was obtained from laboratory reports and calculated using the Chronic Kidney Disease Epidemiology Collaboration (CKD-EPI) equation (20). The primary exposure variable was UHR, calculated as UA (mg/dL) divided by HDL-C (mg/dL) × 100%. To ensure consistency in ratio calculation, glucose, lipid parameters, creatinine, and UA were converted to mg/dL before statistical analysis.

#### 2.2.3 AoAC assessment

The primary outcome was AoAC, assessed using standard chest radiographs. AoAC severity was independently evaluated by two trained observers who were blinded to all clinical information using a validated four-grade ordinal scale (Grades 0–3) (3). Interobserver agreement was assessed using quadratic weighted kappa statistics, and discrepancies between the two observers were resolved by consensus. For subsequent analyses, patients were categorized into a non-severe AoAC group (Grades 0–1) and a severe AoAC group (Grades 2–3).

#### 2.2.4 Statistical analysis

All statistical analyses were performed using R software (version 4.3.2). Continuous variables were tested for normality using the Shapiro–Wilk tests. Normally distributed variables are presented as mean ± standard deviation (SD) and were compared using Student's t-test, whereas non-normally distributed variables are presented as median (interquartile range [IQR]) and were compared using the Mann–Whitney U test. Categorical variables are presented as frequencies (percentages) and were compared using the chi-square test or Fisher's exact test, as appropriate. A two-sided *P* value < 0.05 was considered statistically significant.

Multivariable logistic regression models were constructed to evaluate the association between UHR and severe AoAC. Results are presented as odds ratios (ORs) with 95% confidence intervals (CIs). Three models were constructed using a stepwise adjustment strategy: Model 1 was unadjusted; Model 2 was adjusted for age, sex, and BMI; and Model 3 was further adjusted for smoking, drinking, hypertension, diabetes mellitus, CKD, CHD, dyslipidemia, antihypertensive medications, glucose-lowering medications, statins, and antiplatelet medications.

Restricted cubic spline (RCS) analysis with four knots placed at the 5th, 35th, 65th, and 95th percentiles of the UHR distribution was performed to evaluate potential nonlinear associations with severe AoAC. Overall and nonlinear associations were assessed using P-overall and P-nonlinear values, respectively. To evaluate the robustness of the primary findings and the potential impact of dichotomizing AoAC, sensitivity analyses were performed using ordinal logistic regression with the original four-grade AoAC scale (Grades 0–3), applying the same covariate adjustment strategy as in the primary analyses.

Receiver operating characteristic (ROC) curve analysis was performed to evaluate the discriminatory performance of UHR and the different regression models, and the areas under the curve (AUCs) were compared using the DeLong test. Subgroup analyses were conducted according to demographic characteristics (sex, age, and BMI), lifestyle factors (smoking and drinking), comorbidities (hypertension, diabetes mellitus, CHD, dyslipidemia, arrhythmia, HF, CKD, and stroke), renal function (eGFR), and medication use. Interaction terms were included to evaluate potential effect modification.

### 2.3 Mendelian randomization analysis

A two-sample MR design was used to evaluate the potential causal effects of UA and HDL-C on AAC. MR analysis relies on three core assumptions: (1) the selected genetic variants are robustly associated with the exposure; (2) the variants are independent of potential confounding factors; and (3) the variants influence the outcome exclusively through the exposure of interest.

#### 2.3.1 GWAS data sources

Genetic instruments for UA were derived from the Open GWAS database (ebi-a-GCST90018977), including 343,836 individuals of European ancestry. Summary statistics for HDL-C were obtained from the GWAS dataset GCST90497348, comprising 434,646 individuals of predominantly European ancestry. Genetic associations with AAC were obtained from the GWAS dataset GCST90134614, which included 31,786 and 41,203 individuals in the discovery and replication cohorts, respectively. All GWAS datasets were derived predominantly from individuals of European ancestry, thereby minimizing potential bias due to population stratification. As the research relied solely on publicly available summary-level data. Therefore, no additional ethical approval was required.

#### 2.3.2 Instrumental variable selection

Single nucleotide polymorphisms (SNPs) that were significantly associated with the exposures were chosen as instrumental variables at a genome-wide significance level of *P* < 5 × 10⁻⁸. To ensure independence of the selected SNPs, linkage disequilibrium (LD) clumping was performed with an r^2^ threshold < 0.001 within a 10,000-kb window, using the European ancestry reference panel from the 1000 Genomes Project. Clumping analyses were implemented locally via PLINK for HDL-C and on the OpenGWAS platform for UA, with identical parameters applied (21, 22). Exposure and outcome datasets were harmonized to align effect alleles, and palindromic SNPs with intermediate allele frequencies were removed to avoid strand ambiguity.

#### 2.3.3 Instrument strength

The proportion of phenotypic variance explained (R^2^) by each genetic instrument was calculated based on the SNP–exposure association estimates using the following formula:

$$R^{2}=\frac{2\beta^{2}EAF(1-EAF)}{2\beta^{2}EAF(1-EAF)+2NEAF(1-EAF)SE^{2}}$$

where *β* represents the SNP–exposure effect estimate, SE denotes the corresponding standard error, EAF is the effect allele frequency, and N is the sample size of the exposure GWAS (23). instrument strength was assessed using the F-statistic (F = *β*^2^/SE^2^). SNPs with F-statistics > 10 were considered sufficiently strong to minimize weak instrument bias (24, 25).

#### 2.3.4 Statistical analysis and sensitivity analysis

The inverse-variance weighted (IVW) method under a random-effects model was used as the primary MR approach to estimate the potential causal effects of UA and HDL-C on AAC. Complementary MR methods, including MR-Egger regression, the weighted median estimator, the simple mode, and the weighted mode, were additionally applied to evaluate the robustness of the findings. Horizontal pleiotropy was assessed using the MR-Egger intercept test, heterogeneity was evaluated using Cochran's Q statistic, and potential outlier SNPs were identified using the MR-PRESSO method. Leave-one-out and single-SNP analyses were further performed to evaluate the influence of individual genetic variants on the overall causal estimates.

Steiger directionality filtering was not performed because the AAC GWAS summary statistics did not contain complete sample size information required for this analysis. All analyses were performed using the TwoSampleMR package in R (version 4.3.2), and statistical significance was assigned to a two-sided *P* value below 0.05.

### 2.4 Network-based analysis

An integrative network-based analysis was performed to investigate the potential molecular mechanisms underlying the association between UA and VC. Putative targets of UA were retrieved from the ChEMBL, STITCH, and SwissTargetPrediction databases, while VC-related genes were obtained from the GeneCards and Online Mendelian Inheritance in Man (OMIM) databases. After removing duplicate entries, the overlapping targets between UA and VC were identified as candidate genes for subsequent analyses.

Using the STRING database (version 11.5), a protein–protein interaction (PPI) network of the intersecting targets was constructed (Homo sapiens; minimum interaction score > 0.4), and Cytoscape (version 3.9.1) was used for network visualization and topological analysis. Hub genes were identified using the CytoHubba plugin based on degree centrality. Functional enrichment analyses, including Gene Ontology (GO) and Kyoto Encyclopedia of Genes and Genomes (KEGG) pathway analyses, were performed using the clusterProfiler package in R, with an adjusted *P* value < 0.05 considered statistically significant.

## 3 Results

### 3.1 Baseline characteristics of the study population

**Figure 1:**
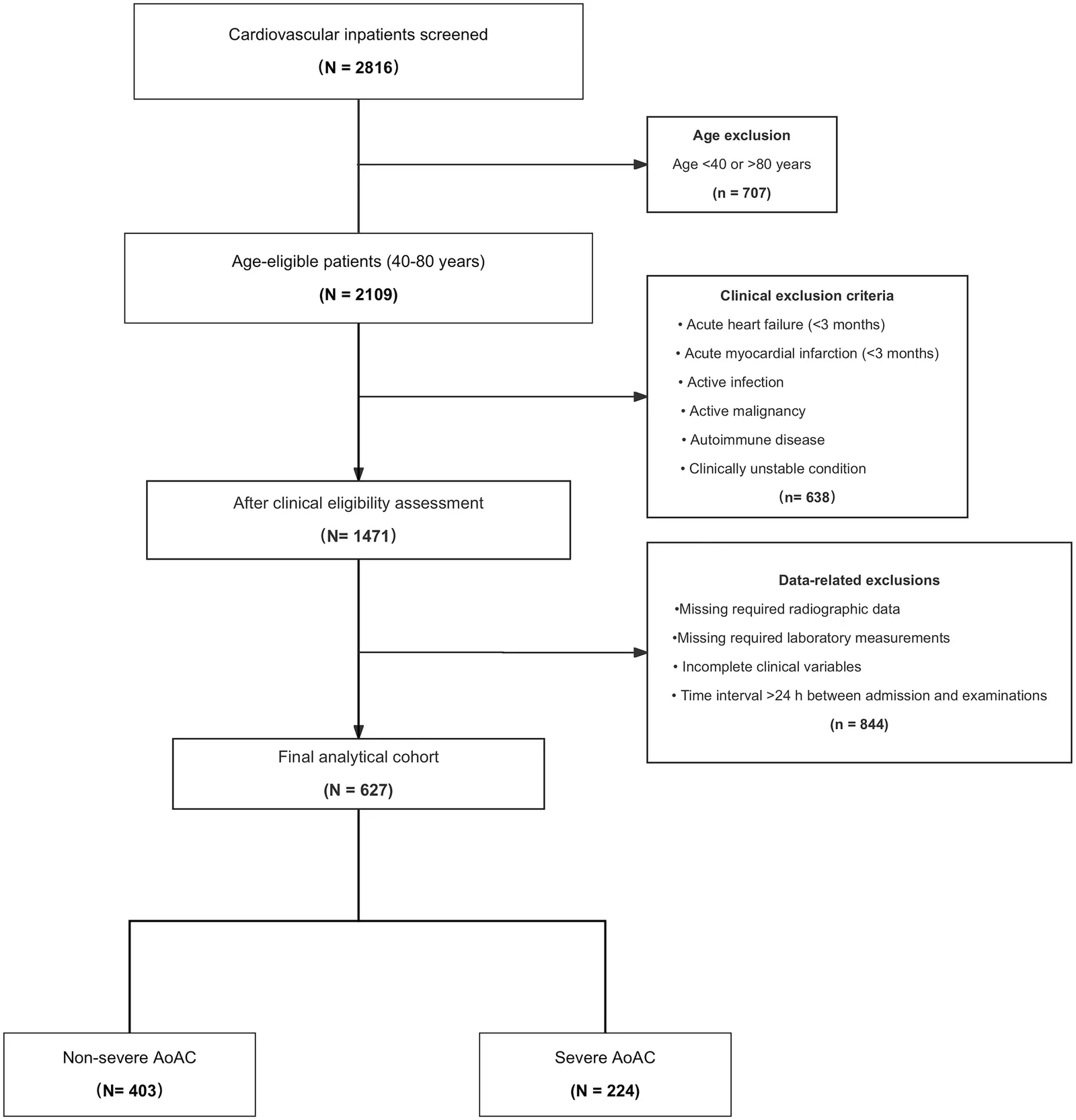
Flowchart of study population selection and grouping. Consecutive cardiovascular inpatients admitted between September 2024 and August 2025 were screened for eligibility. Patients aged 40–80 years with available chest radiographs and fasting laboratory measurements obtained within 24 h of admission were eligible. After applying the predefined clinical and data-related exclusion criteria, 627 patients were included in the final analysis. Aortic arch calcification (AoAC) was assessed using a validated four-grade ordinal scale, and participants were categorized into non-severe AoAC (Grades 0–1, *n* = 403) and severe AoAC (Grades 2–3, *n* = 224) groups.

A total of 627 eligible cardiology inpatients were included in the final analysis after the predefined eligibility screening and exclusion process (Figure 1). Based on chest radiography, 224 (35.7%) patients were classified into the severe AoAC group (Grades 2–3), whereas 403 (64.3%) were categorized into the non-severe group (Grades 0–1). Patients with severe AoAC were significantly older than those without (median age: 71.00 [67.00, 75.00] vs. 63.00 [55.00, 69.00] years; *P* < 0.001), while sex distribution and BMI were comparable between groups (both *P* > 0.05). The prevalence of hypertension, diabetes mellitus, coronary heart disease, chronic kidney disease, and previous stroke was significantly higher in the severe AoAC group (all *P* < 0.01; Table 1).

**Table 1:** Baseline characteristics.

Variable	Non-severe (*n* = 403)	Severe (*n* = 224)	*P* value
Gender (*n*,%)			
Female	205 (50.9%)	121 (54.0%)	0.501
Male	198 (49.1%)	103 (46.0%)	
Age(years)	63.00 [55.00, 69.00]	71.00 [67.00, 75.00]	<0.001
BMI(kg/m^2^)	24.01 [21.64, 26.41]	23.96 [21.71, 26.96]	0.861
Smoking	91 (22.6%)	50 (22.3%)	1.000
Drinking	41 (10.2%)	16 (7.1%)	0.263
Hypertension	274 (68.0%)	184 (82.1%)	<0.001
Diabetes	89 (22.1%)	78 (34.8%)	<0.001
CHD	183 (45.4%)	155 (69.2%)	<0.001
Dyslipidemia	172 (42.7%)	75 (33.5%)	0.030
Arrhythmia	90 (22.3%)	58 (25.9%)	0.364
HF	37 (9.2%)	20 (8.9%)	1.000
CKD	29 (7.2%)	34 (15.2%)	0.002
Stroke history	58 (14.4%)	53 (23.7%)	0.005
Antihypertensives	172 (42.7%)	155 (69.2%)	<0.001
Antidiabetics	68 (16.9%)	71 (31.7%)	<0.001
Statins	88 (21.8%)	79 (35.3%)	<0.001
Antiplatelets	67 (16.6%)	77 (34.4%)	<0.001
WBC(10^9^/L)	6.76 [5.63, 7.92]	6.62 [5.68, 8.04]	0.895
Hemoglobin(g/dL)	13.40 [12.50, 14.40]	13.20 [12.40, 14.10]	0.059
Platelet(10^9^/L)	231.00 [191.50, 276.00]	226.00 [190.00, 272.00]	0.446
FPG(mg/dL)	95.40 [86.40, 109.80]	99.00 [90.00, 117.00]	0.004
Creatinine(mg/dL)	0.78 [0.67, 0.97]	0.89 [0.71, 1.04]	<0.001
UA(mg/dL)	5.55 [4.49, 6.58]	5.94 [5.07, 7.18]	<0.001
eGFR(mL/min/1.73 m^2^)	90.40 [76.20, 99.10]	79.35 [63.50, 89.00]	<0.001
Triglyceride(mg/dL)	110.71 [79.27, 157.21]	110.71 [81.26, 156.10]	0.989
Total cholesterol(mg/dL)	175.18 [146.17, 204.18]	163.57 [135.44, 194.70]	0.002
HDL-C(mg/dL)	43.70 [36.74, 52.01]	41.18 [34.80, 49.59]	0.023
LDL-C(mg/dL)	99.38 [74.83, 125.68]	89.91 [64.77, 117.94]	0.005
UHR(%)	12.63 [9.18, 17.49]	14.62 [10.51, 19.20]	<0.001

Participants comprised hospitalized Chinese cardiovascular patients aged 40–80 years who were recruited from Guangzhou Red Cross Hospital, Guangzhou, China, between September 2024 and August 2025. The study was approved by the Ethics Committee of Guangzhou Red Cross Hospital (Approval No. 2025-243-02).

BMI, body mass index; CHD, coronary heart disease; HF, heart failure; CKD, chronic kidney disease; WBC, white blood cell count; FPG, fasting plasma glucose; UA, uric acid; eGFR, estimated glomerular filtration rate; HDL-C, high-density lipoprotein cholesterol; LDL-C, low-density lipoprotein cholesterol; UHR, uric acid to HDL cholesterol ratio.

Data are presented as mean ± standard deviation (SD), median (interquartile range, IQR), or number (percentage, %), as appropriate.

*P* values were calculated using Student's t test, Mann–Whitney U test, or chi-square test, depending on data distribution and variable type.

The use of antihypertensive, antidiabetic, lipid-lowering, and antiplatelet medications was more frequent in patients with severe AoAC (all *P* < 0.001; Table 1), accompanied by lower total cholesterol and LDL-C levels, likely reflecting increased statin use. Serum uric acid levels were higher and HDL-C levels were lower in the severe group, resulting in a significantly elevated UHR (14.62 [10.51, 19.20] vs. 12.63 [9.18, 17.49], *P* < 0.001; Table 1). Interobserver agreement for AoAC grading was excellent(quadratic weighted *κ* = 0.914, 95% CI: 0.897–0.932).

### 3.2 Association between UHR and AoAC severity

Multivariable logistic regression analyses were performed using three models: Model 1 (crude), Model 2 (adjusted for age, sex, and BMI), and Model 3 (further adjusted for smoking status, drinking status, hypertension, diabetes mellitus, chronic kidney disease (CKD), coronary heart disease [CHD], dyslipidemia, and medication use, including antihypertensive drugs, antidiabetic drugs, statins, and antiplatelet agents). UHR was significantly associated with severe AoAC across all three models (Table 2). In Model 1, each 1-unit increase in UHR was associated with a 3.9% increase in the odds of severe AoAC (OR=1.039, 95% CI: 1.014–1.065, *P* = 0.003), and this association remained significant after adjustment in Model 2 (OR=1.058, 95% CI: 1.028–1.092, *P* < 0.001) and persisted in the fully adjusted Model 3 (OR=1.050, 95% CI: 1.017–1.085, *P* = 0.003).

**Table 2:** Association between UHR and severe aortic arch calcification.

Model	Variable	OR (95% CI)	*P* value
Model 1	UHR	1.039 (1.014–1.065)	0.003
Model 2	UHR	1.058 (1.028–1.092)	<0.001
Model 3	UHR	1.050 (1.017–1.085)	0.003

UHR, uric acid to high-density lipoprotein cholesterol ratio; OR, odds ratio; CI, confidence interval.

Model 1 was unadjusted.

Model 2 was adjusted for age, sex, and body mass index (BMI).

Model 3 was further adjusted for smoking, drinking, hypertension, diabetes mellitus, chronic kidney disease (CKD), coronary heart disease (CHD), dyslipidemia, antihypertensive medications, glucose-lowering medications, statins, and antiplatelet medications.

To evaluate the robustness of the primary findings and the potential impact of dichotomizing AoAC severity, a sensitivity analysis was performed using the original four-category AoAC grades (0–3) as an ordinal outcome. Ordinal logistic regression produced results consistent with those of the primary binary logistic regression analysis. In the fully adjusted model, higher UHR levels remained associated with increasing AoAC severity (OR=1.054, 95% CI: 1.024–1.083, *P* < 0.001; Supplementary Table S1).

### 3.3 Relationship between UHR and AoAC

**Figure 2:**
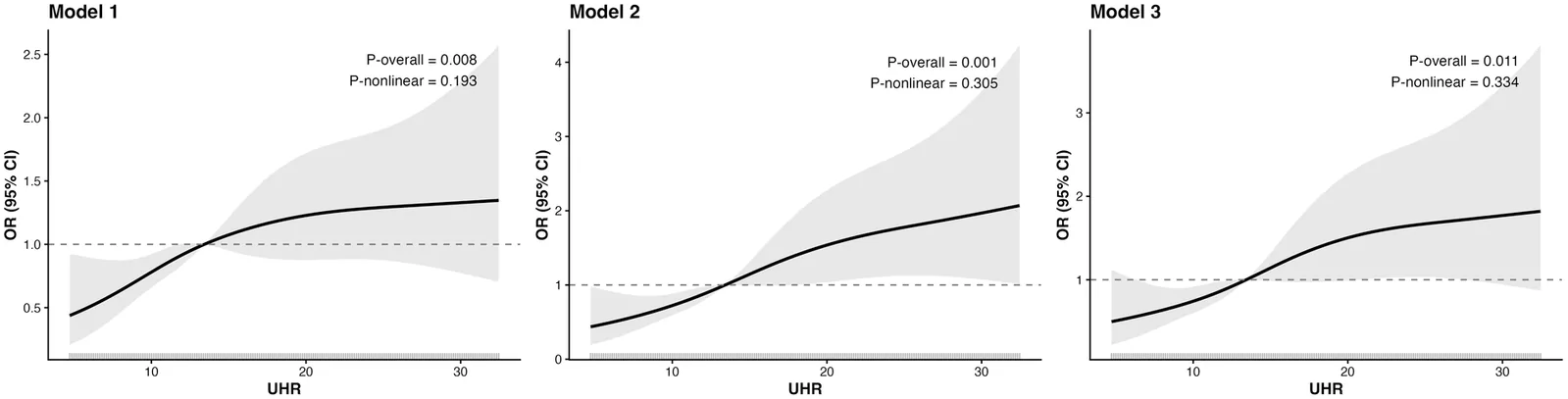
Restricted cubic spline analysis of the association between uric acid-to-high-density lipoprotein cholesterol ratio (UHR) and severe aortic arch calcification (AoAC). Restricted cubic spline models with four knots placed at the 5th, 35th, 65th, and 95th percentiles of the UHR distribution were used to evaluate the association between UHR and severe AoAC. Model 1 was unadjusted; Model 2 was adjusted for age, sex, and BMI; and Model 3 was further adjusted for smoking, drinking, hypertension, diabetes mellitus, CKD, CHD, dyslipidemia, antihypertensive medications, glucose-lowering medications, statins, and antiplatelet medications. Solid lines represent odds ratios (ORs), and shaded areas indicate 95% confidence intervals. Overall and nonlinear associations were assessed using P-overall and P-nonlinear values, respectively.

**Figure 3:**
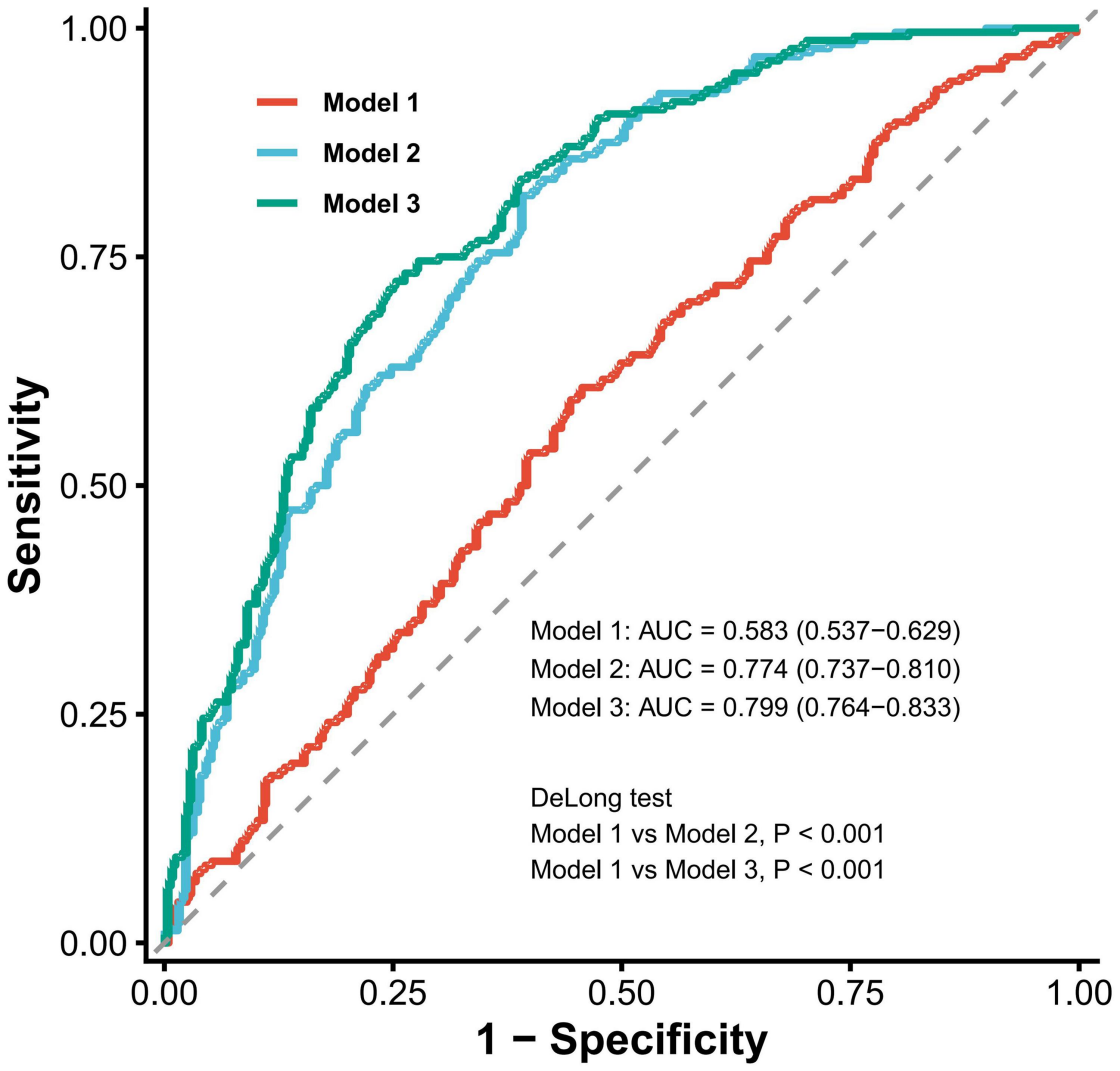
Receiver operating characteristic (ROC) curves for predicting severe aortic arch calcification (AoAC). ROC curves were used to evaluate the discriminatory performance of the three regression models for severe AoAC. Model 1 was unadjusted; Model 2 was adjusted for age, sex, and BMI; and Model 3 was further adjusted for smoking, drinking, hypertension, diabetes mellitus, CKD, CHD, dyslipidemia, antihypertensive medications, glucose-lowering medications, statins, and antiplatelet medications. The area under the curve (AUC) was calculated for each model, and differences between AUCs were assessed using the DeLong test.

RCS analysis suggested a linear association between UHR and severe AoAC across all models, with no evidence of a nonlinear relationship in Model 1 (*P* for non-linearity = 0.193), Model 2 (*P* = 0.305), or Model 3 (*P* = 0.303; Figure 2). In the fully adjusted model, the overall association remained statistically significant (*P* for overall association = 0.010). ROC curve analysis showed that the discriminatory performance improved progressively across the three models, with the AUC increased from 0.583 (95% CI: 0.537–0.629) in Model 1 to 0.774 (95% CI: 0.737–0.810) in Model 2 and 0.799 (95% CI: 0.764–0.833) in Model 3 (Figure 3). The AUC of Model 3 was significantly higher than that of Model 1 (DeLong test *P* < 0.001).

### 3.4 Subgroup and interaction analyses

**Figure 4:**
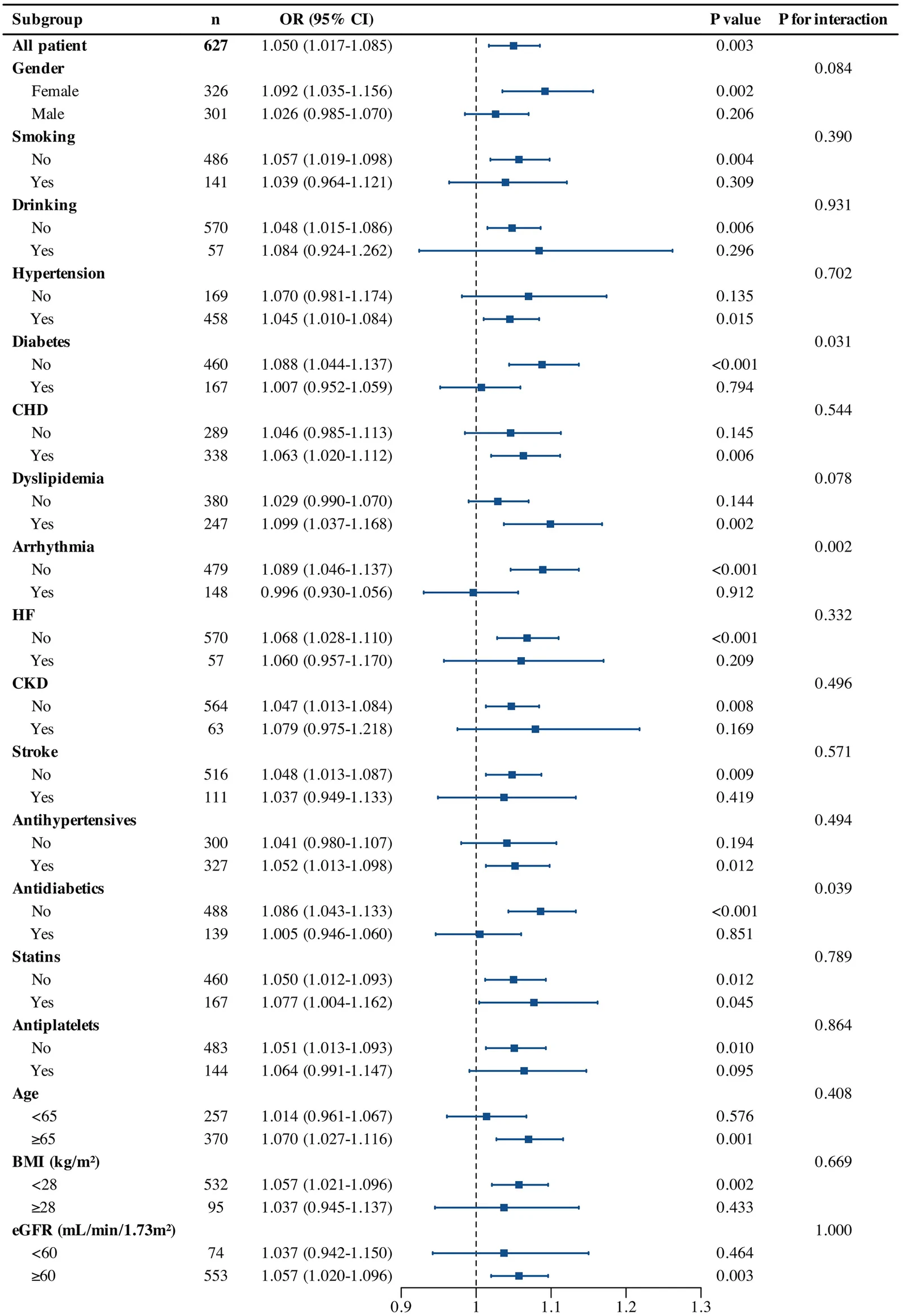
Subgroup and interaction analyses of the association between uric acid-to-high-density lipoprotein cholesterol ratio (UHR) and severe aortic arch calcification (AoAC). Subgroup analyses were performed using the fully adjusted logistic regression model (Model 3), which was adjusted for smoking, drinking, hypertension, diabetes mellitus, CKD, CHD, dyslipidemia, antihypertensive medications, glucose-lowering medications, statins, and antiplatelet medications, in addition to age, sex, and BMI. Odds ratios (ORs) and 95% confidence intervals (CIs) are presented for each subgroup. *P* values for interaction were calculated to evaluate potential effect modification.

Subgroup analyses were performed using the fully adjusted model (Model 3) (Figure 4). Significant interactions were observed for diabetes (*P* for interaction = 0.031), arrhythmia (*P* for interaction = 0.002), and antidiabetic medication use (*P* for interaction = 0.039). In stratified analyses, the association between UHR and severe AoAC remained significant among patients without diabetes, without arrhythmia, and not receiving antidiabetic treatment, but was not observed in the corresponding subgroups (Figure 4). No significant interactions were identified for the remaining subgroup variables (all *P* for interaction > 0.05).

### 3.5 Mendelian randomization analysis

**Figure 5:**
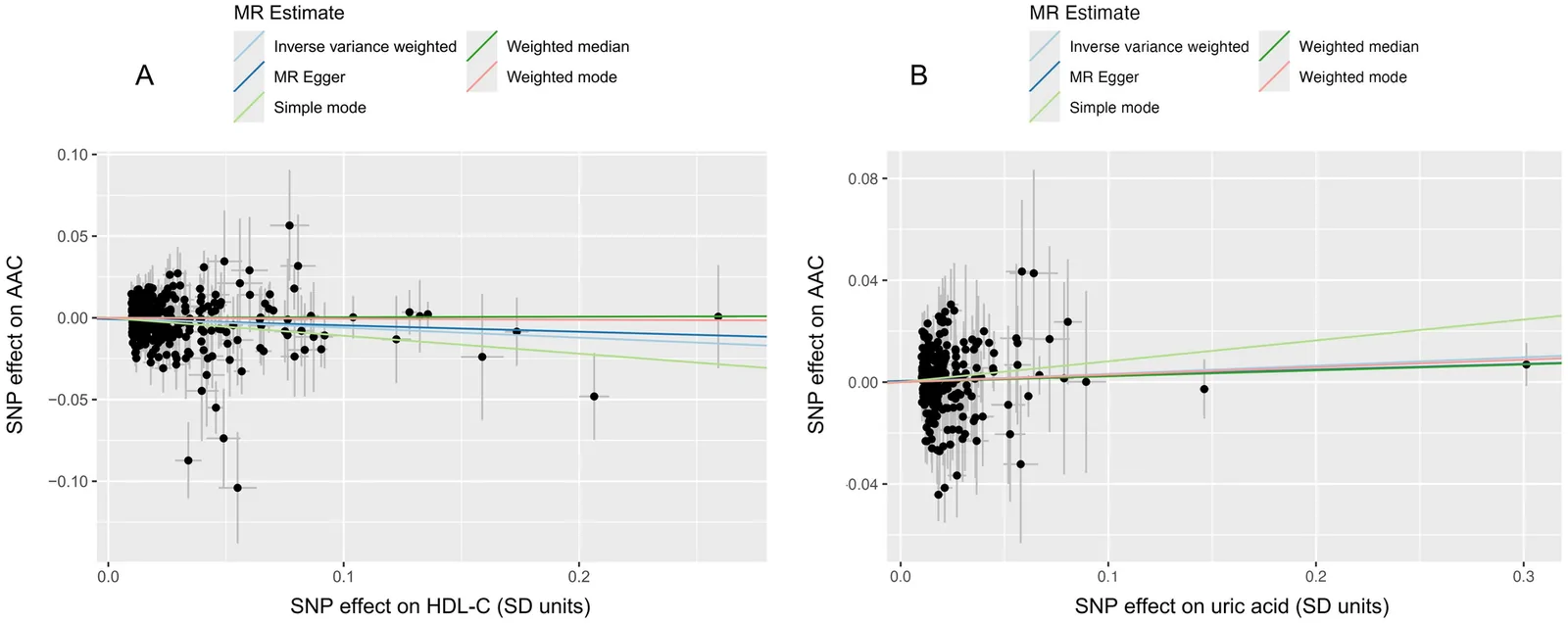
Mendelian randomization (MR) scatter plots for the associations of high-density lipoprotein cholesterol (HDL-C) and uric acid (UA) with abdominal aortic calcification (AAC). **(A)** Scatter plot of SNP effects on HDL-C versus SNP effects on AAC. **(B)** Scatter plot of SNP effects on UA versus SNP effects on AAC. Each point represents a single nucleotide polymorphism (SNP). The slopes of the fitted lines represent causal effect estimates obtained using different MR methods, including inverse variance weighted (IVW), MR-Egger, weighted median, weighted mode, and simple mode.

A total of 244 SNPs for UA and 358 SNPs for HDL-C were selected as instrumental variables, and all instruments had F-statistics > 10. AAC was used as the outcome in a two-sample MR analysis(Figures 5A,B). No significant causal association was observed between genetically predicted UA levels and AAC (IVW: *β* = 0.033, SE = 0.023, *P* = 0.166), and consistent null results were obtained using the MR-Egger, weighted median, simple mode, and weighted mode methods. In contrast, genetically predicted HDL-C levels were inversely associated with AAC in the primary IVW analysis (*β* = −0.061, SE = 0.020, *P* = 0.003); however, this association was not consistently supported by the complementary MR methods (Table 3). Detailed information on the GWAS datasets and genetic instruments is provided in Supplementary Tables S2–S4.

**Table 3:** Mendelian randomization estimates of UA and HDL-C in relation to abdominal aortic calcification.

Exposure	nsnp	Method	*β* (SE)	*P* value
UA	244	IVW	0.033 (0.023)	0.166
		MR-Egger	0.022 (0.031)	0.479
		Weighted median	0.023 (0.028)	0.403
		Simple mode	0.082 (0.110)	0.456
		Weighted mode	0.029 (0.024)	0.231
HDL-C	358	IVW	−0.061 (0.020)	0.003
		MR-Egger	−0.039 (0.031)	0.216
		Weighted median	0.003 (0.033)	0.917
		Simple mode	−0.110 (0.074)	0.139
		Weighted mode	−0.006 (0.035)	0.870

UA, uric acid; HDL-C, high-density lipoprotein cholesterol; IVW, inverse variance weighted; SE, standard error; SNP, single nucleotide polymorphism.

Effect estimates represent the change in abdominal aortic calcification per one standard deviation increase in genetically predicted exposure.

Sensitivity analyses showed no evidence of horizontal pleiotropy according to the MR-Egger intercept test (all *P* > 0.05), whereas Cochran's *Q* test indicated significant heterogeneity for both UA and HDL-C (Supplementary Table S5). The MR-PRESSO global test was significant for both exposures. One outlier SNP was identified for UA, but the outlier-corrected estimate remained non-significant (distortion test *P* = 0.879), whereas no outlier SNPs were detected for HDL-C (Supplementary Table S6). Leave-one-out and single-SNP analyses suggested that the overall estimates were not driven by any individual SNP (Supplementary Figures S1–S6).

### 3.6 Network-based analysis

**Figure 6:**
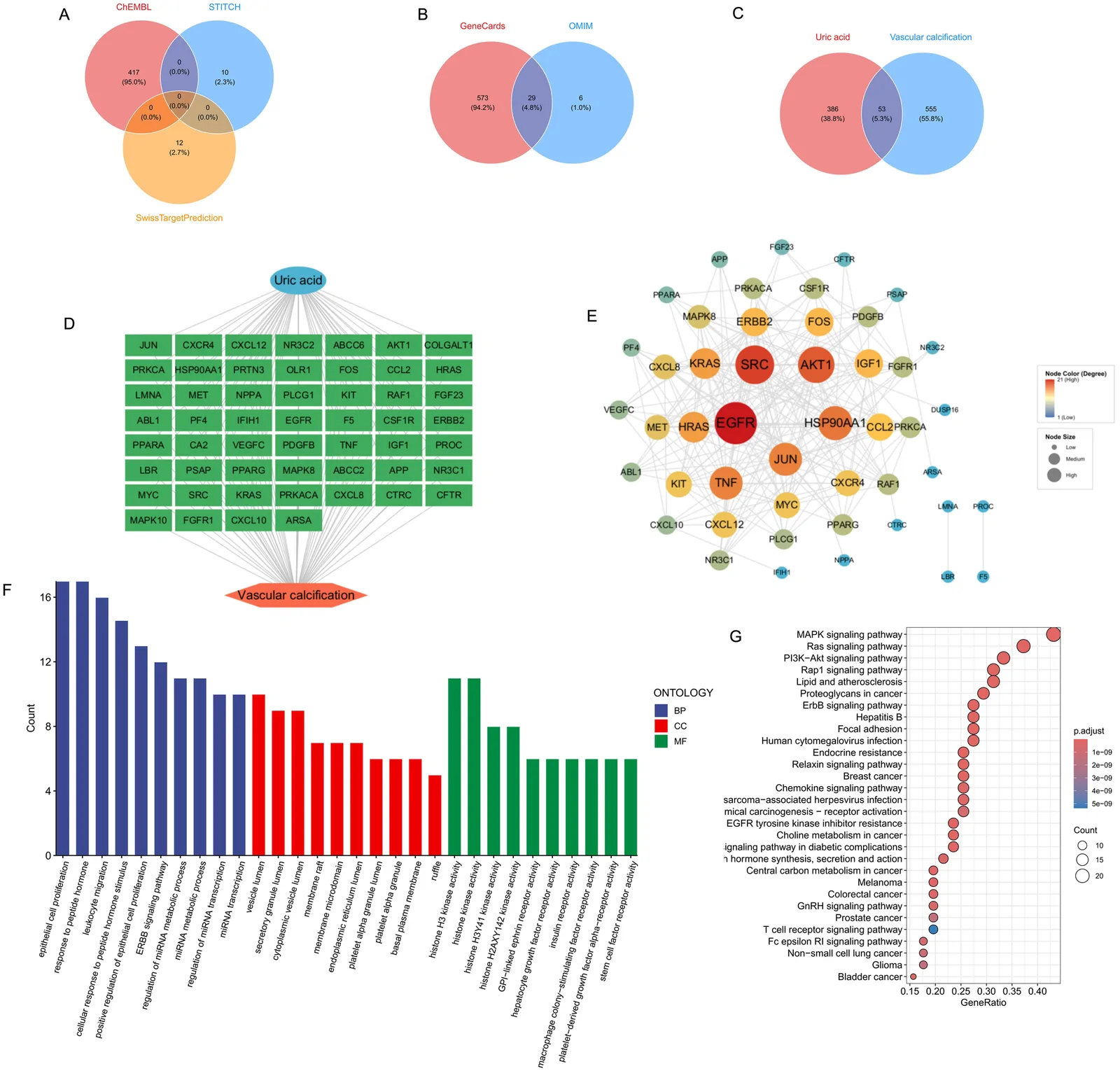
Network-based analysis of potential targets linking uric acid to vascular calcification. **(A)** Venn diagram illustrating the integration of uric acid-related targets retrieved from the ChEMBL, STITCH, and SwissTargetPrediction databases. **(B)** Venn diagram illustrating the integration of vascular calcification-related genes retrieved from the GeneCards and OMIM databases. **(C)** Venn diagram showing the overlap between uric acid-related targets and vascular calcification-related genes after database integration and removal of duplicate entries. A total of 53 overlapping targets were identified and retained for subsequent analyses. **(D)** Target interaction network linking uric acid and vascular calcification through the 53 overlapping targets identified in panel C. **(E)** Protein–protein interaction (PPI) network of the 53 overlapping targets identified in panel C. Node size and color intensity are mapped according to node degree. Larger nodes and warmer colors indicate higher degree values and stronger network connectivity. **(F)** Gene Ontology (GO) enrichment analysis performed using the 53 overlapping targets identified in panel C, including biological process (BP), cellular component (CC), and molecular function (MF). **(G)** Kyoto Encyclopedia of Genes and Genomes (KEGG) pathway enrichment analysis performed using the 53 overlapping targets identified in panel C.

Potential UA-related targets were retrieved from the ChEMBL, STITCH, and SwissTargetPrediction databases. After integration and removal of duplicate entries, a comprehensive set of UA-related targets was obtained (Figure 6A; Supplementary Table S7). Similarly, VC-related genes were retrieved from the GeneCards and OMIM databases and integrated into a unified gene set (Figure 6B; Supplementary Table S8). Intersection analysis identified 53 overlapping targets between UA and VC, which were considered candidate targets potentially linking UA to VC and were selected for subsequent network analyses (Figure 6C; Supplementary Table S9). The constructed UA–target–VC network illustrated the interactions among these shared targets (Figure 6D). PPI network analysis identified several highly connected hub genes, including EGFR, SRC, AKT1, TNF, and JUN (Figure 6E). Functional enrichment analysis indicated that these targets were mainly involved in biological processes related to cell proliferation, inflammatory response, and signal transduction (Figure 6F). KEGG pathway analysis demonstrated significant enrichment in the MAPK, PI3K–Akt, Ras, and lipid and atherosclerosis signaling pathways (Figure 6G).

## 4 Discussion

This study integrated evidence from clinical association analysis, MR analysis, and network-based approaches to investigate the relationship between UHR and VC. Although these analytical layers addressed different aspects of the same research question, they were intended to provide complementary rather than mutually validating evidence. The clinical analysis demonstrated an association between UHR and AoAC, whereas the MR analysis offered component-level genetic evidence related to UA and HDL-C. In addition, network-based analyses suggested potential molecular pathways involved in VC. Taken together, these findings suggest that the association between UHR and VC is unlikely to be explained by the direct effect of a single biological component. Instead, UHR may reflect the combined influence of multiple metabolic and inflammatory processes associated with VC.

In this cohort of clinically stable cardiology inpatients, higher UHR levels were associated with greater severity of AoAC after multivariable adjustment, with an approximately linear relationship observed across the range of UHR values, consistent with previous population-based analyses (14). Although our study included clinically stable cardiovascular inpatients rather than a general population, the consistent direction of association across different study settings may support the robustness of this observational finding. However, the discriminative performance of UHR alone was modest, suggesting that its clinical utility may be improved when incorporated into multivariable risk models rather than used as a standalone indicator. The strength of this association varied across clinical subgroups, with a more pronounced relationship observed among individuals without diabetes or atrial fibrillation. This pattern may reflect differences in the underlying pathophysiological context. In diabetes, chronic hyperglycemia and insulin resistance contribute to vascular calcification through mechanisms such as advanced glycation and sustained oxidative stress (26, 27). Atrial fibrillation is associated with systemic inflammation, endothelial dysfunction, and vascular remodeling (28), which may attenuate the relative contribution of metabolic indices such as UHR.

An inverse association between genetically predicted HDL-C levels and AoAC was observed in the IVW analysis, but this finding was not consistently supported across sensitivity methods, precluding a definitive causal interpretation. Previous MR studies have also reported heterogeneous findings regarding the causal role of HDL-C in cardiovascular outcomes (29). This inconsistency may partly reflect the complex biological properties of HDL (30). In addition, HDL functionality, rather than HDL-C concentration alone, may be more relevant to cardiovascular risk, as suggested by more consistent associations of HDL particle measures with VC and cardiovascular outcomes (31, 32), as well as experimental evidence showing that specific HDL-associated proteins such as ANGPTL3 can modulate cholesterol efflux and anti-inflammatory functions (33). Taken together, these findings suggest that HDL-C concentration alone may not fully capture the biological heterogeneity of HDL, and the observed association should therefore be interpreted with caution.

In contrast, there was no notable link found between genetically predicted UA levels and AAC. Previous MR studies have reported complex and sometimes inconsistent associations between UA and outcomes (34). Both experimental and clinical data show that UA is linked to oxidative stress, inflammatory processes, and endothelial dysfunction, but these effects are often embedded within broader metabolic disturbances, including hypertension, insulin resistance, and chronic inflammation (35, 36). Accordingly, the influence of UA on VC may depend on the overall metabolic context rather than representing an isolated effect, and its predictive value as a single biomarker may therefore be limited. Therefore, the absence of a significant genetic association should not necessarily be interpreted as evidence against a potential biological role of UA in VC.

The apparent discrepancy between the observational and MR findings may therefore reflect the different biological levels captured by these approaches. The clinical analysis evaluated UHR as a composite metabolic marker that integrates information from both UA and HDL-C within a specific metabolic context, whereas the MR analysis examined the genetic effects of each component separately. Given that the biological effects of UA and HDL-C are influenced by broader metabolic and inflammatory conditions, their individual genetic associations may not fully capture the complex processes reflected by UHR. Consequently, the association observed between UHR and vascular calcification may represent the cumulative influence of multiple interconnected pathways rather than the direct effect of any single component alone.

The association identified in this research between UHR and VC may reflect the interplay of metabolic and inflammatory processes. VC is a complex pathological process involving oxidative stress, chronic inflammation, and dysregulated lipid metabolism, which are closely interconnected (37). At the systems level, network-based analyses suggested that uric acid–related targets were involved in pathways associated with inflammation and vascular remodeling, particularly MAPK and PI3K–Akt signaling. These pathways are associated with processes like vascular inflammation, endothelial dysfunction, and the phenotypic transformation of vascular smooth muscle cells, all contributing to the progression of VC (38, 39). Furthermore, crucial hub genes were pinpointed in the protein-protein interaction network, including TNF, AKT1, and EGFR, have been reported to participate in inflammatory signaling and vascular remodeling. TNF acts as a central mediator of vascular inflammation and calcification, while AKT1 and EGFR are involved in cell survival, proliferation, and osteogenic differentiation of vascular smooth muscle cells (40, 41). These findings provide a systems-level context for the observed clinical associations. However, as these results are derived from bioinformatics analyses, they should be considered hypothesis-generating and require further experimental validation. Together with the clinical and genetic findings, these results provide complementary evidence supporting the hypothesis that UHR reflects a composite metabolic and inflammatory milieu rather than the direct effect of a single biological pathway.

From a clinical perspective, UHR represents a readily available and cost-effective biomarker that integrates metabolic and inflammatory information. Due to its link with VC found in this study, UHR might assist in identifying people at greater risk of calcification, particularly in settings where imaging-based assessment is not routinely available, and may serve as an additional indicator for risk stratification in clinical practice. These findings, however, should be approached with caution. The current results do not establish a causal relationship, and the clinical applicability of UHR remains preliminary. Further validation in prospective and multicenter studies is required before its potential role as part of multivariable risk assessment strategies can be established.

This study has several limitations. First, the clinical analysis was based on a single-center, cross-sectional cohort, precluding causal inference and limiting the generalizability of the findings. Ethnicity information was not collected as part of this retrospective study and therefore could not be reported, which may further limit the generalizability of the findings to populations of different ethnicities. Second, AoAC was assessed using chest radiographs rather than CT, and different VC phenotypes (AoAC and AAC) were evaluated across the clinical and MR analyses, which may have introduced heterogeneity. Third, because no GWAS data are currently available for UHR, the MR analysis was limited to its individual components (UA and HDL-C) rather than UHR itself. In addition, Steiger directionality filtering could not be performed because complete sample size information was unavailable in the AAC GWAS dataset. Finally, the network-based analyses were exploratory and derived from bioinformatics predictions, requiring further experimental validation. Future multicenter prospective studies are warranted to validate these findings.

## 5 Conclusion

In conclusion, UHR was significantly associated with AoAC, a clinically accessible marker of VC, in clinically stable cardiology inpatients. Genetic analyses did not provide consistent evidence supporting a direct causal role of the individual components of UHR, whereas network-based analyses suggested potential biological pathways linking UA to VC. Overall, these findings suggest that UHR may serve as a composite metabolic biomarker reflecting the combined influence of multiple metabolic and inflammatory processes associated with VC.

## Data Availability

The raw data supporting the conclusions of this article will be made available by the authors, without undue reservation.
