# Supplementary material for: Exploring the relationship between uric acid-to-HDL cholesterol ratio and vascular calcification: evidence from clinical, genetic, and network-based analyses
Source: Front Cardiovasc Med. 2026 Aug 12;13:1877627. doi: 10.3389/fcvm.2026.1877627 (PMC13506826; doi:10.3389/fcvm.2026.1877627)
Supplement: Supplementary file 1 [file Datasheet1.pdf]

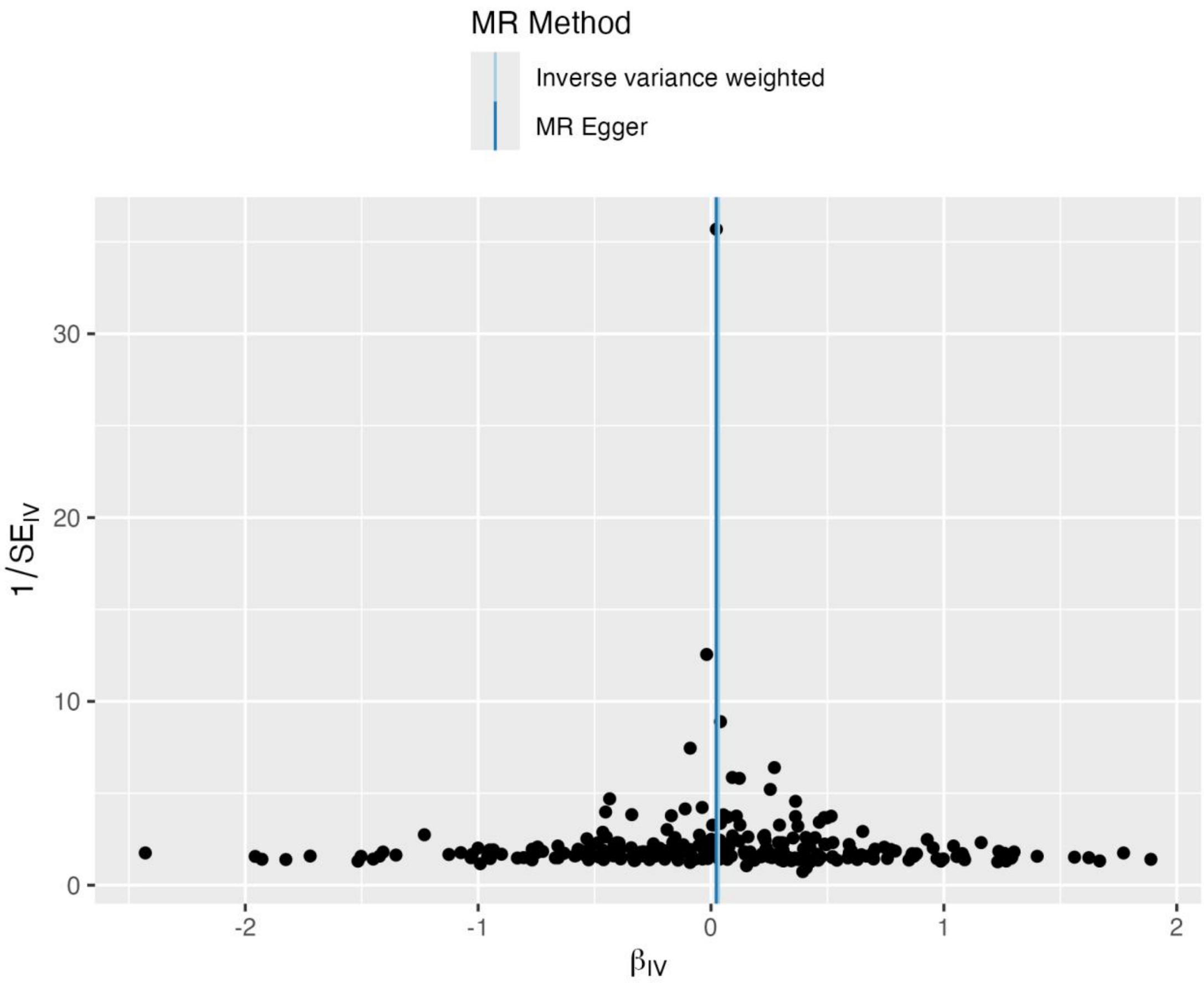

Supplementary Figure S1. Funnel plot for Mendelian randomization analysis of serum uric acid (UA) and abdominal aortic calcification (AAC). Each point represents an individual single nucleotide polymorphism (SNP). The overall symmetrical distribution of SNP-specific estimates around the inverse-variance weighted (IVW) effect suggests no obvious evidence of directional pleiotropy.

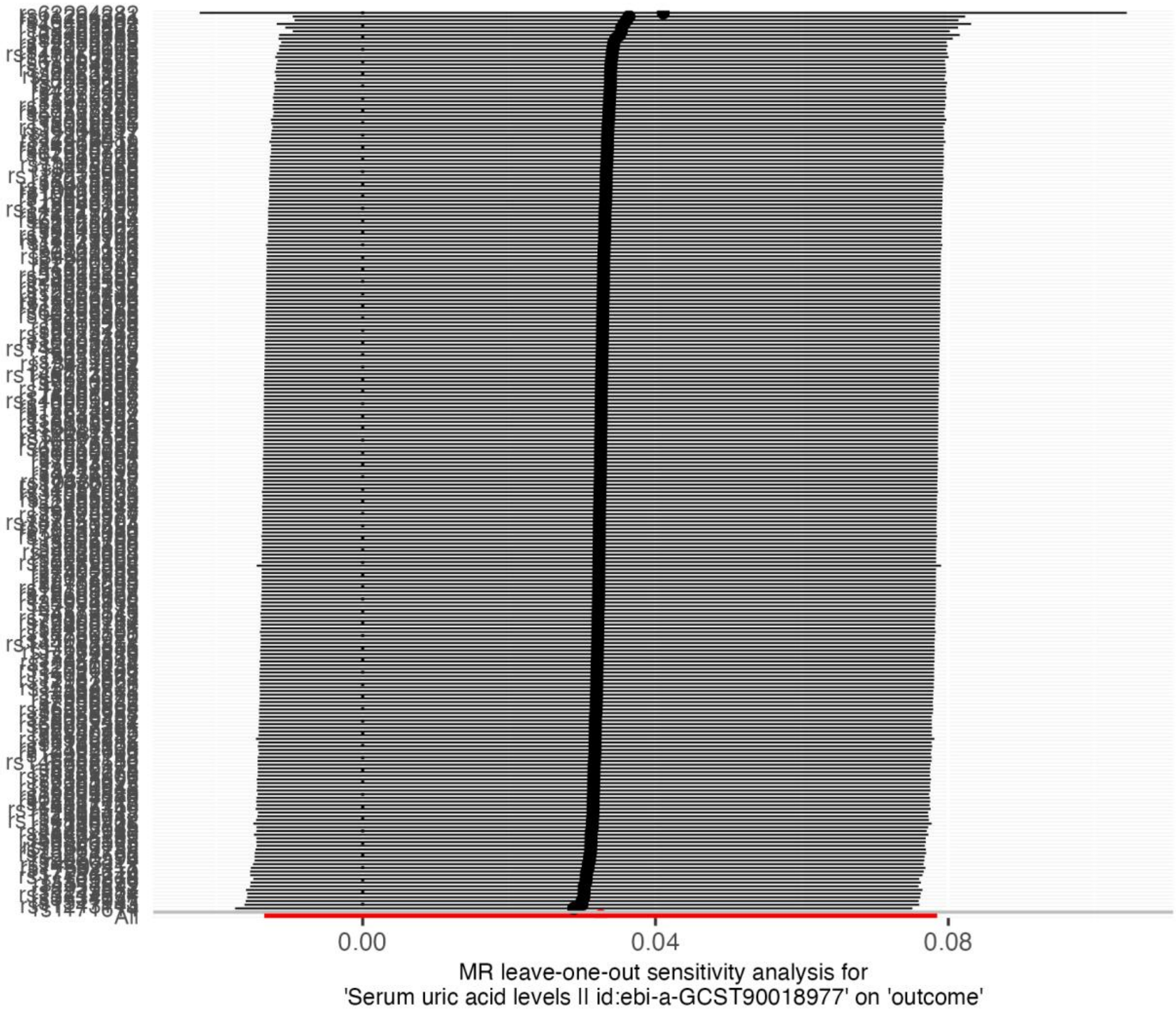

Supplementary Figure S2. Leave-one-out sensitivity analysis for Mendelian randomization analysis of serum uric acid (UA) and abdominal aortic calcification (AAC). Each point represents the causal estimate obtained after sequentially removing one single nucleotide polymorphism (SNP) at a time. Sequential removal of individual SNPs did not materially alter the overall effect estimate, indicating that the observed result was not driven by any single instrumental variable.

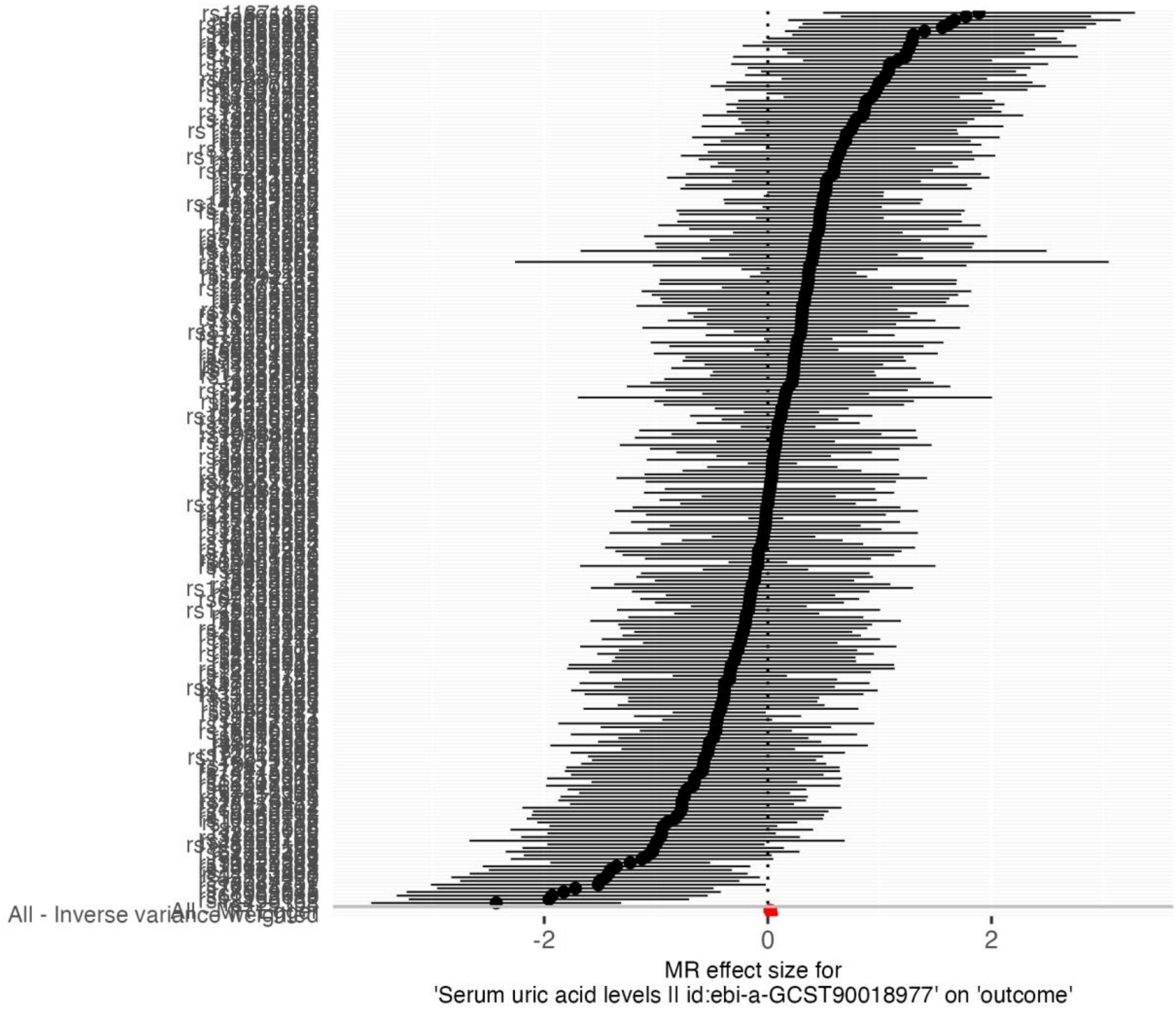

Supplementary Figure S3. Forest plot of Mendelian randomization estimates for serum uric acid (UA) and abdominal aortic calcification (AAC). Each line represents the causal estimate for an individual single nucleotide polymorphism (SNP) with its corresponding 95% confidence interval. Most confidence intervals crossed the null value, consistent with the overall MR findings showing no statistically significant causal association between UA and AAC.

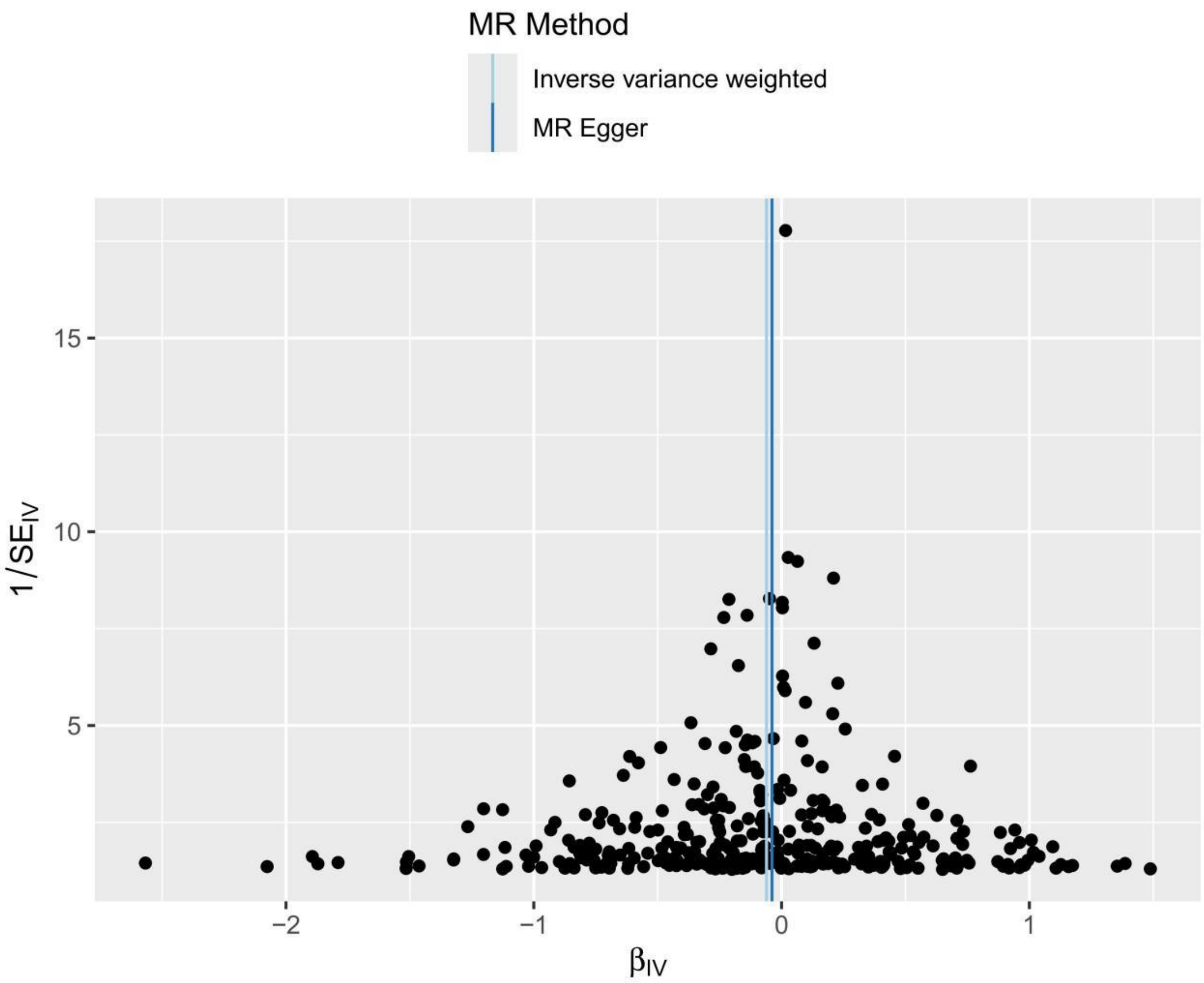

Supplementary Figure S4. Funnel plot for Mendelian randomization analysis of high-density lipoprotein cholesterol (HDL-C) and abdominal aortic calcification (AAC). Each point represents an individual single nucleotide polymorphism (SNP). The overall symmetrical distribution of SNP-specific estimates around the inverse-variance weighted (IVW) effect suggests limited evidence of directional pleiotropy.

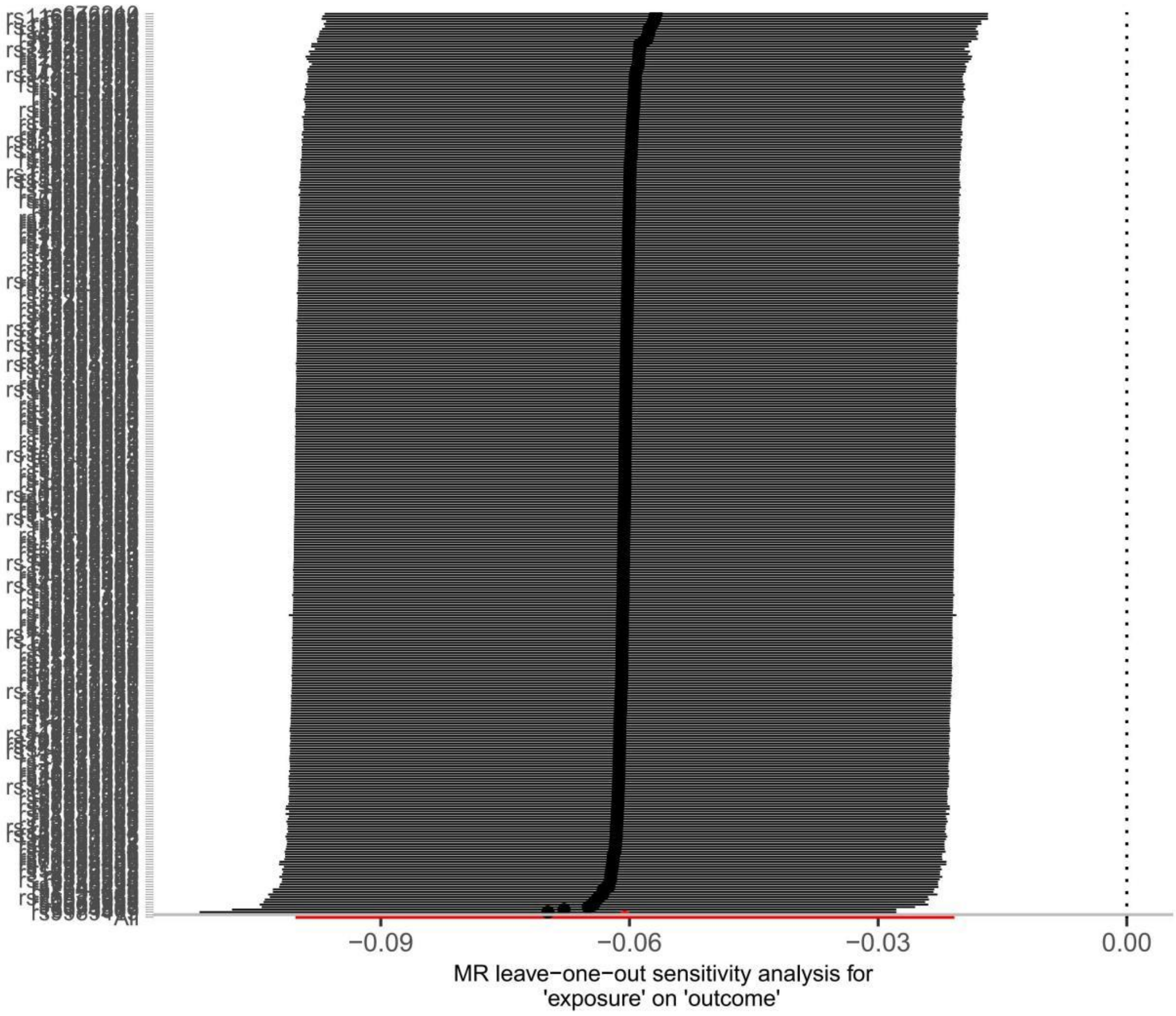

Supplementary Figure S5. Leave-one-out sensitivity analysis for Mendelian randomization analysis of high-density lipoprotein cholesterol (HDL-C) and abdominal aortic calcification (AAC). Each point represents the causal estimate obtained after sequentially removing one single nucleotide polymorphism (SNP) at a time. Exclusion of any single SNP did not substantially alter the overall causal estimate, indicating that the inverse association observed in the inverse-variance weighted (IVW) analysis was not driven by any individual instrumental variable.

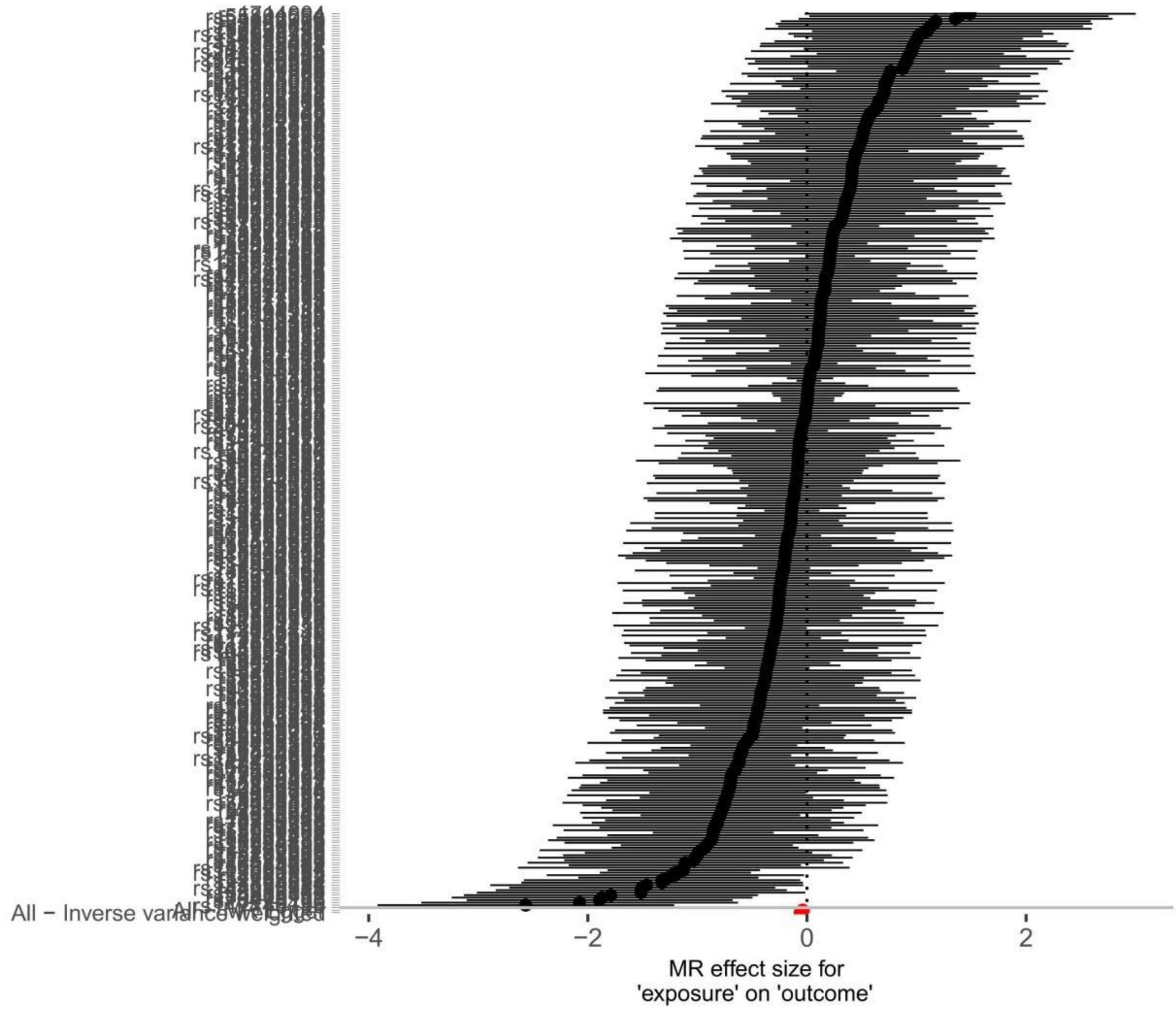

Supplementary Figure S6. Forest plot of Mendelian randomization estimates for high-density lipoprotein cholesterol (HDL-C) and abdominal aortic calcification (AAC). Each line represents the causal estimate for an individual single nucleotide polymorphism (SNP) with its corresponding 95% confidence interval. Although individual SNP effect estimates varied, the overall direction of effect was generally consistent with the inverse-variance weighted (IVW) result, suggesting a potential inverse association between genetically predicted HDL-C levels and AAC.
